# Editorial: New approaches in Brain-Machine Interfaces with implants

**DOI:** 10.3389/fnins.2024.1485472

**Published:** 2024-09-26

**Authors:** Vahid Salari, Rodney O'Connor, Serafim Rodrigues, Daniel Oblak

**Affiliations:** ^1^Department of Physics and Astronomy, University of Calgary, Calgary, AB, Canada; ^2^Department of Chemical Engineering, University of Polytechnique Montreal, Montreal, QC, Canada; ^3^Mines Saint-Etienne, Centre CMP, Département BEL, Gardanne, France; ^4^Basque Center for Applied Mathematics (BCAM), Bilbao, Spain

**Keywords:** microfluidics, 3D-printed, neurotechnologies, Brain Computer Interface (BCI), neurohacking, implant, ECoG, LFP polarity

Brain-Machine Interfaces (BMIs) or Brain-Computer Interfaces (BCIs) open new possibilities in neuroscience, creating direct interaction between the brain and external devices (Salari et al., 2022). These systems can help individuals with disabilities by translating brain signals into commands. Despite recent progress in BCI with implants, the field still encounters many technological and biological obstacles. For instance, they are working on developing a much smaller, more powerful implant that can be placed in the brain after a simple surgery which could bring control to people with paralysis. However, it is anticipated that progress in the field of brain implants has been hampered by a combination of technological and biological factors, such as the limited understanding of the long-term behavior of implants, unreliability of devices, and biocompatibility of the implants among others. Invasive BCI requires surgery to implant electrodes under the scalp for communicating brain signals. The main advantage is to provide a more accurate reading; however, its downside includes side effects from the surgery. After the surgery, scar tissues may form which can make brain signals weaker.

This Research Topic of Frontiers in Neuroscience investigates new findings related to some challenges in the field and advancing BCI capabilities.

## 1 Printable devices for neurotechnologies

The first article reviews the growing field of printable electronics for neurotechnology (Matta et al.), which proposes rapid prototyping, scalability, and cost-effectiveness. The study features the development of printable neuro-probes and microelectrode arrays for recording and stimulating neuronal activity. These devices, made from biocompatible and flexible materials, aim to enhance our understanding of brain functions and improve treatments for neurological disorders. The potential of printable electronics to facilitate more effective communication between the brain and external systems sets the stage for the transformative advancements discussed in this Research Topic.

Printable chips for neurotechnologies include microelectrode arrays (MEAs) that record and stimulate neuronal activity for high-resolution monitoring of brain signals. Electrocorticography (ECoG) arrays enable the recording of electrical activity from the brain's surface, useful for both research and clinical applications. Additionally, 3D-printed neural probes facilitate minimally invasive insertion into brain tissue for targeted stimulation and recording of neural circuits. Bioelectronic interfaces integrate printed electrodes with biological systems for applications like drug delivery and real-time physiological monitoring, while flexible sensors conform to the brain's surface, providing a comfortable interface for long-term monitoring and interaction with neural tissues.

## 2 Photonic neural probes with 3D-printed microfluidics

Building on the theme of new materials and methods, the second paper focuses on photonic neural probes integrated with 3D-printed microfluidics (Mu et al.). These probes combine optogenetic stimulation and neurochemical delivery, proposing precise control over neural circuits and real-time monitoring of neurochemical changes. The study shows successful neurochemical injections and localized photoactivation in brain tissue, showcasing the potential of these advanced probes to develop therapeutic strategies and improve BCI performance.

The Research Topic focuses on the development and optimization of neural probes for enhanced neurochemical delivery and monitoring. Key findings include the variation in transmission of grating emitters, which ranged from 27 to 20 dB, with a median of 22 dB due to alignment drift. Compensation techniques such as laser power modulation and MEMS mirror adjustments were employed to address these issues. Additionally, the integration of 3D-printed microfluidic structures allows for neurochemical injection without altering existing fabrication processes, leading to successful uncaging experiments in fixed brain tissue that demonstrated localized photoactivation of caged dyes. The study investigates the potential of these advanced neural probes in neuroscience research for more precise and effective methods of studying neural activity and drug delivery.

## 3 ECoG devices: assessing long-term biocompatibility

The third study investigates the long-term biocompatibility of Parylene HT -ITO ECoG devices through immunohistochemical evaluations in mice (Madarász et al.). The Research Topic discloses an initial astroglial response that diminishes over time and a transient reduction in cortical thickness, which normalizes in later assessments. Neuronal density was lower on the implanted side only at the last evaluation, suggesting a potential stabilization of neuronal health, while cortical thickness was reduced in the initial assessments but returned to normal by the last time point, showing a recovery of cortical structure. The Research Topic discusses the suitability of Parylene HT/ITO ECoG devices for chronic applications, focusing on the importance of evaluating biocompatibility to ensure the safe and effective use of implantable devices in BCI applications. The result shows the long-term biocompatibility of the Parylene HT/ITO ECoG devices, suggesting their feasibility for chronic use in neuroscience applications.

## 4 Understanding LFP polarity for enhanced BCI performance

The fourth article investigates local field potentials (LFPs) and current source density (CSD) in the primary visual cortex (V1) of macaque monkeys (Khodaei et al.). By analyzing how stimulus size and eccentricity influence neural activity across cortical layers, the study provides an understanding of the neural mechanisms underlying visual processing. Improved understanding of LFPs and CSD can enhance the accuracy and effectiveness of BCIs, aiding individuals with disabilities in controlling devices and facilitating communication.

## 5 The world of neurohackers: ethical and practical implications

The final paper explores the practices and perspectives of neurohackers, individuals who engage with neurotechnology for personal enhancement and development (Seyfried et al.). Through qualitative interviews, the study reveals diverse motivations and ethical concerns associated with neurohacking. Examples such as RFID chip implants for personal use and BCIs for device control illustrate the innovative and sometimes controversial nature of neurohacking. The Research Topic investigates the need for a deeper understanding of the ethical, safety, and societal implications as this field continues to evolve.

## 6 Conclusion

The research presented in this Research Topic of Frontiers in Neuroscience features the dynamic and multifaceted nature of BCI technologies. From the rapid prototyping capabilities of printable electronics to the precision of photonic neural probes and the ethical considerations of neurohacking, these studies focus on considerable developments toward overcoming current limitations. As we continue to explore and transform, these advancements suggest paving the way for more effective, minimally invasive, and adaptable BCIs, ultimately enhancing the quality of life for individuals with neurological conditions and disabilities.
